# Diurnal Patterns in Solute Concentrations Measured with In Situ UV-Vis Sensors: Natural Fluctuations or Artefacts?

**DOI:** 10.3390/s20030859

**Published:** 2020-02-06

**Authors:** Suzanne R. Jacobs, Björn Weeser, Mariana C. Rufino, Lutz Breuer

**Affiliations:** 1 Center for international Development and Environmental Research (ZEU), Justus Liebig University, Senckenbergstr. 3, 35390 Giessen, Germany; bjoern.weeser@umwelt.uni-giessen.de (B.W.); lutz.breuer@umwelt.uni-giessen.de (L.B.); 2 Institute for Landscape Ecology and Resources Management (ILR), Justus Liebig University, Heinrich-Buff-Ring 26, 35392 Giessen, Germany; 3 Lancaster Environment Centre, Lancaster University, Lancaster LA1 4YQ, UK; m.rufino1@lancaster.ac.uk; 4 Centre for International Forestry Research (CIFOR), c/o World Agroforestry Centre, United Nations Avenue, Gigiri, 00100 Nairobi, Kenya

**Keywords:** nitrate, dissolved organic carbon, spectrophotometer, high-frequency data

## Abstract

In situ spectrophotometers measuring in the UV-visible spectrum are increasingly used to collect high-resolution data on stream water quality. This provides the opportunity to investigate short-term solute dynamics, including diurnal cycling. This study reports unusual changes in diurnal patterns observed when such sensors were deployed in four tropical headwater streams in Kenya. The analysis of a 5-year dataset revealed sensor-specific diurnal patterns in nitrate and dissolved organic carbon concentrations and different patterns measured by different sensors when installed at the same site. To verify these patterns, a second mobile sensor was installed at three sites for more than 3 weeks. Agreement between the measurements performed by these sensors was higher for dissolved organic carbon (*r* > 0.98) than for nitrate (*r* = 0.43–0.81) at all sites. Higher concentrations and larger amplitudes generally led to higher agreement between patterns measured by the two sensors. However, changing the position or level of shading of the mobile sensor resulted in inconsistent changes in the patterns. The results of this study show that diurnal patterns measured with UV-Vis spectrophotometers should be interpreted with caution. Further work is required to understand how these measurements are influenced by environmental conditions and sensor-specific properties.

## 1. Introduction

The continuous development of water quality sensors has led to a transition from studying long-term trends and seasonal patterns using the time series of monthly or weekly grab samples to the investigation of highly dynamic phenomena, such as storm events and diurnal patterns, using high-frequency in situ measurements. In the past, such events were studied through intensive sampling campaigns, but these studies were often of short duration (e.g., 2–12 days) [1,2,3,4,5,6]. With the currently available technology and decreasing costs, in situ sensors are more frequently used for longer periods, ranging from months, e.g., [7,8] to several years, e.g., [9,10,11]. Although large amounts of data present challenges regarding storage, processing, and analysis [12], longer term datasets provide an opportunity for detailed investigations of hydrological and biogeochemical processes in dynamic systems, especially in remote areas [13,14,15]. Compared to terrestrial ecosystems, where processes such as primary productivity and respiration are highly correlated to climate variables, aquatic ecosystems are more complex. This is a result of seasonal variation in light supply for photosynthesis through seasonal changes in shading by riparian vegetation, temporal changes in autotroph biomass due to floods and droughts, and allochthonous inputs of detritus and organic matter [16]. Long-term studies improve our understanding of the potential effect of land use and climate change on river metabolism and the delivery of water-related ecosystem services [16].

Many terrestrial and in-stream biogeochemical processes are driven by the incidence of solar energy, resulting in distinct patterns on a diel, i.e., a 24-hour timescale. The diel change in solar energy can result in diel patterns in stream temperature, especially in shallow, wide and less shaded streams. Additionally, photosynthetic activity is driven by solar radiation, influencing the concentration of dissolved oxygen (DO) and carbon dioxide (CO_2_) in the stream [17]. In addition, biological activity can result in diel patterns in, for example, dissolved organic carbon (DOC) [6,18,19], phosphorus [6,18,20], and nitrate [20,21,22] through uptake and respiration. The study of diurnal fluctuations is one of the subjects for which the use of in situ sensors is particularly suitable, since current technology allows measuring at intervals of seconds or minutes.

Spectrophotometers measuring in the UV-visible spectrum, also referred to as UV-Vis sensors, can be used to investigate diel patterns for DOC and nitrate. These sensors use algorithms to calculate solute concentrations based on absorbance at a specific wavelength or multiple wavelengths. Although the use of in situ UV-Vis sensors presents several challenges, such as biofouling [23], local calibration [24,25], and power supply [24,26], numerous studies have used such sensors in the field [20,21,27,28,29,30]. In most of these studies, grab samples analyzed in the laboratory were used to validate data recorded by the sensor [24,31]. This sampling, however, does not allow to check the validity of high-frequency patterns, such as diurnal fluctuations.

We deployed four UV-Vis sensors (spectro::lyser, s::can Messtechnik GmbH, Vienna, Austria) to understand nitrate dynamics in streams draining different land use types (tropical montane forest, smallholder agriculture and commercial tea plantations) in the South West Mau region in western Kenya [9]. Diurnal patterns in the nitrate concentration differed between sites and seasons (i.e., rainy season and dry season), suggesting an influence of land use and seasonality on in-stream biogeochemical processes. However, abrupt changes in these diurnal patterns were observed when the position of a sensor was adjusted to facilitate measurements during very low flows. Furthermore, different sensors recorded different patterns at the same site. These observations led to the suspicion that some of these ‘diurnal patterns’ could be artefacts, rather than the manifestation of biological processes. To assess the validity of the observed diurnal patterns, we used a mobile set-up, whereby a second UV-Vis sensor was installed next to an existing monitoring system. The mobile sensor was installed parallel to the fixed UV-Vis sensor for at least two weeks. Afterwards, we shaded or changed the position of the mobile sensor to a different depth and/or orientation for at least another week to investigate whether these changes influenced the measurements.

The article aims to present evidence to challenge the interpretation of diurnal patterns in nitrate and DOC concentrations measured by in situ UV-Vis sensors in tropical headwater streams with low solute concentrations. A combination of rapidly changing environmental conditions, e.g., intense sunlight, fluctuations in water level, stream temperature, and turbidity, could result in unexpected artefacts in the data. These have, to our knowledge, not been documented in previous studies. Yet, this information is essential to guide other users of in situ UV-Vis sensors in the interpretation of their data. Although the results of our experiment and data analysis are not conclusive, we provide explanations on potential causes for the inconsistencies in diurnal patterns, especially when solute concentrations are low. We propose how data collected with in situ UV-Vis sensors can still be used to increase the understanding of hydrological and biogeochemical processes. Furthermore, we make suggestions on methods that can be used to further investigate this issue in field and laboratory experiments.

## 2. Materials and Methods

### 2.1. Study Area

This study was carried out in the South West Mau region in western Kenya, part of the largest remaining indigenous tropical montane forest in East Africa. The outlets of three sub-catchments (27–36 km^2^) within the 1021 km^2^ Chemosit catchment were instrumented (Table 1), following a nested catchment approach. Each sub-catchment drains an area characterized by one of the dominant land use types in the region: tropical montane rainforest (NF), smallholder agriculture (SHA), and commercial tea and tree plantations (TTP). The outlet of the Chemosit catchment, referred to as the main catchment (OUT), was instrumented as well. Elevation in the study area ranges from 1715 m a.s.l. to 2932 m a.s.l. Soils are classified as humic Nitisols [32]. The geology is characterized by phonolitic nephelinites in the upper part and phonolites in the lower part of the catchment [33,34]. The annual precipitation is 1988 ± 328 mm for the years 1905 to 2014 at 2100 m a.s.l. [35]. A more detailed description of the study area is provided in Jacobs et al. [35].

### 2.2. Instrumentation

Automatic measurement stations were installed at the outlets of the three sub-catchments (NF, SHA and TTP) in October 2014 and at the main catchment (OUT) in April 2015. Each station consists of a radar-based water level sensor (VEGAPULS WL61, VEGA Grieshaber KG, Schiltach, Germany) and a UV-Vis spectrophotometer (spectro::lyser, s::can Messtechnik GmbH, Vienna, Austria), measuring turbidity, total and dissolved organic carbon (TOC, DOC), and nitrate (NO_3_-N) with a wavelength range of 220 to 720 nm, a resolution of 2.5 nm, and a 5 mm optical path length. In addition, the sensor measures stream temperature. The measurement range and accuracy for nitrate, when measured with this path length, are 0 to 60 mg N L^−1^ and ±2% + 0.2 mg N L^−1^, respectively, according to the sensor documentation. The measurement range for DOC is 0 to 84 mg C L^−1^, but no information on accuracy is provided by the manufacturer. Measurements are taken every 10 min. An automated cleaning system uses bursts of pressurized air to remove any particles from the sensor window before each measurement. In addition, the sensors are cleaned manually on a weekly to bi-weekly basis to reduce the influence of more persistent fouling.

**Figure A1:**
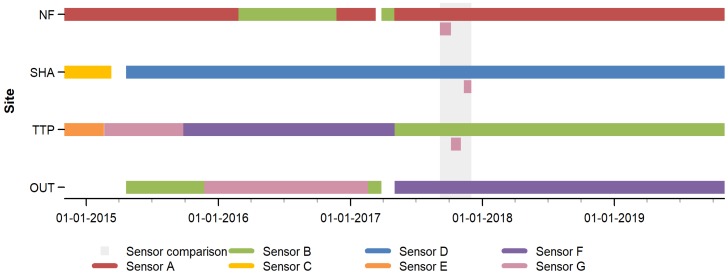
Deployment period of spectro::lyser sensors at the four measurement sites.

The sensor manufacturer provides no specific requirements for the orientation of the sensor. The UV-Vis sensors at NF, SHA, and OUT were installed at a 45° angle at the downstream face of a concrete block in the riverbank. This position protects the sensor from obstruction and damage by woody debris and stones during high flows. Because a similar construction was not possible at TTP, the sensor was installed vertically against the rocky riverbank in a fast-flowing section of the stream. During very low flow (e.g., dry season in February and March), the sensors were mounted in a horizontal position approximately 5 cm above the riverbed. To avoid the trapping of air bubbles from the automated cleaning system at the sensor window, we decided to install the sensor in a way that the measurement gap faces downstream, rather than facing the riverbed. As a consequence, UV radiation from the sun could potentially reach the measurement window during measurements. Due to sensor failure (e.g., energy loss of Xenon lamp, internal dark noise error, corrosion of measurement window), sensors have been replaced and repaired several times at each site (Figure A1). Sensors A, B, and F were rotated between the sites NF, TTP, and OUT on 2 May 2017.

Rainfall was recorded with tipping buckets (Theodor Friedrichs, Schenefeld, Germany, and ECRN-100 high-resolution rain gauge, Decagon Devices, Pullman, WA, USA) as cumulative precipitation in 10-minute intervals (0.2 mm resolution) at nine sites in the study area. To calculate the total rainfall within a (sub-)catchment, each tipping bucket was assigned a weight based on Thiessen polygons. Malfunctioning tipping buckets were temporarily excluded, and the weights of the remaining tipping buckets were adjusted.

### 2.3. Experimental Set-Up

The performance of multiple sensors is best compared by installing all sensors at the same site, but this was logistically not feasible. Instead, an extra sensor (sensor G), referred to as the mobile sensor, was used to test the comparability of patterns recorded by the sensors installed at three of the four measurement stations (fixed sensors). The same sensor had been deployed previously as fixed sensor at TTP and OUT (Figure A1). The mobile sensor was installed at each site for a minimum of three weeks before being moved to another site (Table 2). Installation failed at OUT because of problems with the power supply. The mobile sensor was connected to a control box provided by the manufacturer. This control box was then connected to the 12V power supply and to the data logger (con::cube, s::scan Messtechnik GmbH, Vienna, Austria) for data transmission. The mobile sensor was connected to the pressurized cleaning system with the same cleaning frequency as the fixed sensor. Measurements were taken concurrently by both sensors. The mobile sensor was initially installed in parallel to the fixed sensor. After at least two weeks, either the position of the sensor was changed (depth and/or orientation) or the measurement window of the mobile sensor was shaded (Table 2) to test how this would affect the measurements. Each treatment lasted a minimum of three days.

### 2.4. Data Processing

All data were subjected to a processing protocol, which was previously applied in Jacobs et al. [9]. In summary, time stamps with NA values or those indicated as errors by the internal data logger software (moni::tool, s::can Messtechnik GmbH, Vienna, Austria) were flagged automatically. In addition, observations during field visits were used to manually flag periods with unreliable data due to, e.g., burial by sediment, too low water level or a problem with the automatic cleaning system. The median absolute deviation (MAD) for a rolling window of 16 measurements was used to identify outliers [9,36]. All flagged data were omitted from further analysis. After flagging, data gaps of <6 h were filled using linear interpolation. For easier identification of diurnal patterns, noise in the measured data was removed by applying a rolling mean with a window width of 3 h.

Discharge was estimated using a site-specific rating curve developed using individual discharge measurements over the full range of measured water levels, see Jacobs et al. [9]. During weekly to bi-weekly maintenance visits, 100 mL grab samples were taken from the streams, filtered immediately using <0.45 µm polypropylene filters (KX syringe filter, Kinesis Ltd., St. Neods, UK) and stored frozen until analysis in the laboratory of Justus Liebig University Giessen, Germany. The grab samples were analyzed for nitrates using ion chromatography (ICS-2000, Dionex, Sunnyvale, CA, USA) and for dissolved organic carbon (TOC cube, Elementar Analysensysteme GmbH, Hanau, Germany). These data were used to check and calibrate the nitrate and DOC concentrations recorded by the sensors. For each site, linear regression was used to develop a relationship between the grab samples analyzed in the laboratory and the values measured by the sensors. The reverse of the linear regression equation was then applied to calibrate the full dataset (Appendix B). All data (raw, processed, grab samples for calibration) are available in Jacobs et al. [37].

### 2.5. Data Analysis

A rolling median with a window width of 48 h was applied to the processed dataset to calculate the background concentration of nitrate and DOC. The diurnal patterns were estimated by subtracting the background concentration from the processed dataset, resulting in data representing deviation from background concentration in mg N L^−1^ for nitrate and mg C L^−1^ for DOC.

Because we observed seasonal differences in the occurrence of diurnal patterns, we classified each day into categories representing seasons (dry, transition, rainy). Due to interannual variability in the onset and end of the dry and rainy seasons, we chose to use discharge instead of fixed dates or months as an objective indicator for the different seasons. For each site, sub-daily discharge was aggregated to mean daily discharge (*Q_d_*). We then classified each day into one of three discharge classes: low flow (*Q_d_* ≤ *Q*_70_), medium flow (*Q*_70_ < *Q_d_* ≤ *Q*_30_), and high flow (*Q_d_* > *Q*_30_), representing the dry, transition, and rainy seasons, respectively. *Q*_70_ and *Q*_30_ are the discharge values exceeded on 70% and 30% of the days. The days were further grouped by sensor to investigate potential sensor-specific patterns. At every 10 min of the day, the median and the interquartile range of the deviation from the background concentration were calculated per site, sensor, and discharge class. These data were plotted for a visual investigation of diurnal patterns in nitrate and DOC concentrations in the stream.

The data obtained during the sensor comparison experiment were investigated by comparing the time series of the deviation from the background concentration. Pearson’s correlation coefficients (*r*) were calculated for the deviations measured by the fixed and the mobile sensor for each treatment, as this goodness of fit criteria particularly addresses the correct timing of events.

## 3. Results

### 3.1. Sensor-Specific Patterns

**Figure 1:**
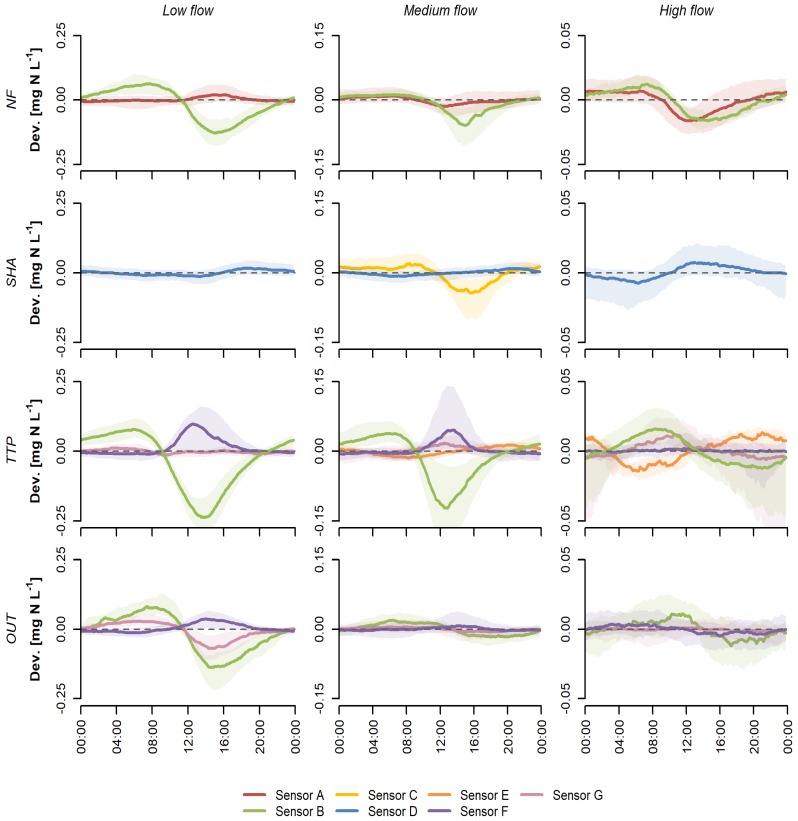
Sensor-specific diurnal patterns for nitrate, expressed as deviations from the background concentration, classified by low flow (*Q_d_* ≤ *Q*_70_), medium flow (*Q*_70_ < *Q_d_* ≤ *Q*_30_), and high flow (*Q_d_* > *Q*_30_) for the four study sites (the tropical montane rainforest (NF), smallholder agriculture (SHA), tea and tree plantations (TTP), and the main catchment (OUT)) in the South West Mau region, Kenya, between November 2014 and October 2019. Note the differences in amplitude in the diurnal patterns between the flow classes. The line indicates the median deviation at a time of day, the shaded area represents the interquartile range of the deviation from the background concentration.

Diurnal patterns in nitrate concentrations in stream water varied between sensors, sites, and discharge classes (Figure 1). The most interesting to compare are the patterns obtained by sensors that were installed at various sites, e.g., sensor B, F, and G at TTP and OUT. Sensor B was also used at NF. Sensors B and F showed similar patterns across sites, although the amplitude differed. However, there was little agreement between patterns recorded with different sensors at the same site, (e.g., sensors B, E, F, and G at TTP). During low flow, sensor B recorded patterns with a fairly large amplitude of up to 0.25 mg N L^−1^, generally showing a low peak around 8 am and minimum between 12 and 4 p.m. In contrast, sensor F generally recorded a maximum of approximately 0.1 mg N L^−1^ between around 12 and 4 p.m. Similar patterns, but with a smaller amplitude (<0.05 mg N L^−1^), were recorded by sensor A at NF during low flow, sensor G at TTP during medium flow, and sensor D during high flow at SHA.

The wide interquartile range of the deviation from the background concentration around the peaks observed with sensor F during low and medium flow at TTP might be a result of temporal changes in the distance between the measurement window and the stream surface. These changes were either caused by natural variation in the water level or by lowering the sensor to enable measuring during very low flows. Conversely, the wider interquartile range during nighttime on high flow days could be caused by the frequent occurrence of rainfall events resulting in a dilution of the nitrate concentration in the stream [9]. The amplitude of most diurnal patterns decreased with increasing discharge, resulting in barely discernible diurnal patterns with mean deviations from the background concentration of <0.02 mg N L^−1^ during high flows. The separation in flow classes also allowed us to assess whether observed patterns depend on the background concentration, as nitrate concentrations increased with discharge at SHA, TTP, and OUT (Table 3). However, we were not able to find a typical pattern at higher concentrations.

**Figure 2:**
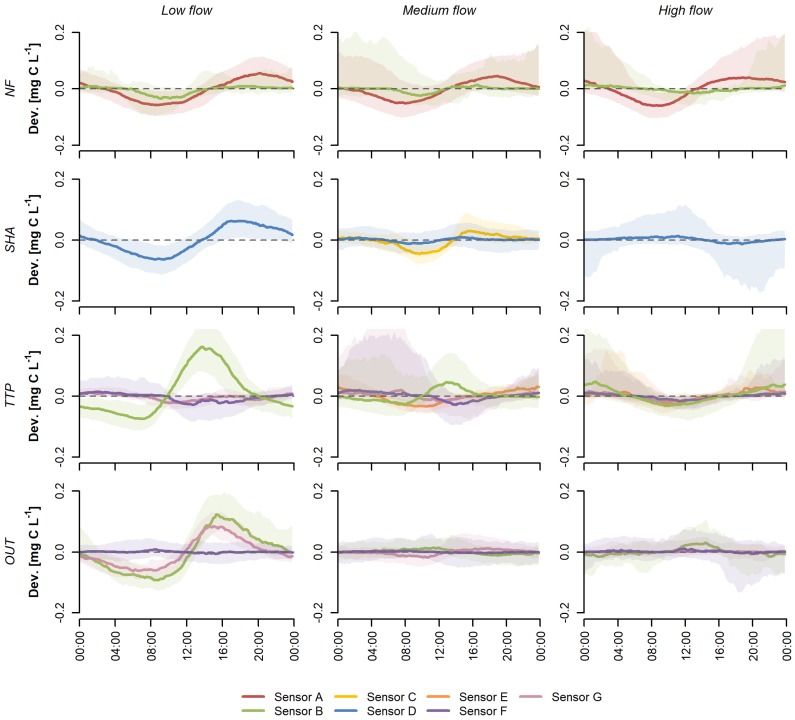
Sensor-specific diurnal patterns for dissolved organic carbon (DOC), expressed as deviations from the background concentration, classified by low flow (*Q_d_* ≤ *Q*_70_), medium flow (*Q*_70_ < *Q_d_* ≤ *Q*_30_), and high flow (*Q_d_* > *Q*_30_) for the four study sites (NF, SHA, TTP and OUT) in the South West Mau region, Kenya, between November 2014 and October 2019. The line indicates the median deviation at a time of day, the shaded area represents the interquartile range of the deviation from the background concentration.

**Figure 3:**
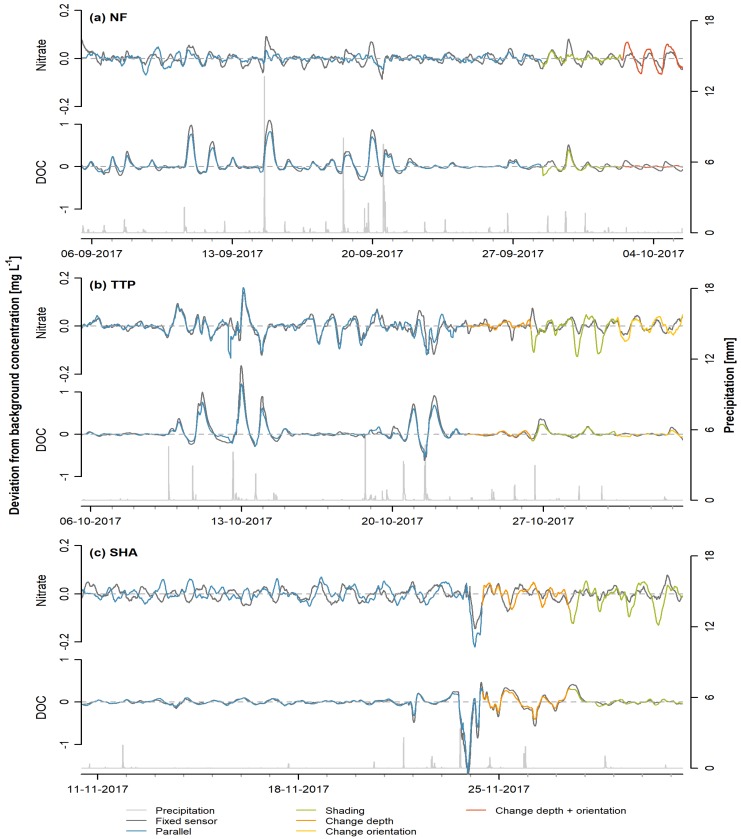
Time series showing cumulative precipitation measured at 10-minute intervals, and the deviation from the background concentration for nitrate and dissolved organic carbon (DOC) during the sensor experiment at the sites (**a**) NF, (**b**) TTP, and (**c**) SHA.

For DOC, the clearest diurnal patterns were observed during low flow at all sites (Figure 2). The background concentration and amplitude of the diurnal patterns were larger than those of nitrate and decreased with increasing discharge. Only at NF were the amplitudes and patterns similar across flow classes when measured with sensor A. The interquartile ranges of the deviation from the background concentration during medium and high flow were quite large, indicating larger variations in the DOC pattern between individual days within each flow class. This is most likely an effect of rainfall events, which affect DOC concentrations more strongly than nitrate concentrations (see, e.g., Figure 3b). Patterns observed in these flow classes should therefore be interpreted with caution. As with nitrate, sensors recorded similar patterns across sites (e.g., sensor B), whereas different patterns were recorded by different sensors at the same site (e.g., OUT; Figure 2).

### 3.2. Sensor Comparison

The small-scale deviations from the background concentration measured with the mobile sensor (sensor G) were in general more comparable to those measured with the fixed sensor for DOC than for nitrate (Figure 3). When the two sensors were in parallel position, TTP showed the highest correlation between deviations for nitrate measured by the two sensors (fixed sensor B; *r* = 0.813, *p* < 0.001). The correlation coefficients were lower for NF (fixed sensor A; *r* = 0.429) and SHA (fixed sensor D; *r* = 0.454). Although the observed patterns for nitrate at NF were largely in phase with the fixed sensor, the mobile sensor at NF did not capture fluctuations of the same amplitude. At SHA, the mobile sensor initially showed a pattern opposite to that recorded by the fixed sensor. After 18 November 2017, the maxima and minima of the diurnal pattern seemed to have shifted by approximately 6 h. This slight phase shift persisted after changing the depth of the mobile sensor and when shading the sensor window.

The deviations from the background concentration measured by both sensors in parallel position (first 2 to 3 weeks) were highly correlated for DOC (*r* > 0.98, *p* < 0.001). DOC concentrations responded strongly to rainfall events, resulting in larger deviations (>0.5 mg C L^−1^) from the background concentration compared to nitrate (Figure 3). Due to frequent rainfall in September 2017, it was difficult to assess whether there were any diurnal patterns in NF. Comparable responses of the DOC concentration to rainfall events were measured by both sensors at all sites. In contrast, patterns were less correlated on drier days in TTP, when the amplitude of the diurnal pattern was low. However, the two sensors recorded comparable diurnal patterns on days without rainfall in SHA.

The change in position or shading of the mobile sensor did not have consistent effects on the diurnal patterns recorded. Shading reduced the amplitude of the nitrate pattern at NF, while lower minima were observed at SHA and TTP. The agreement in the data collected by both sensors during shading was much higher for DOC (*r* > 0.8 at all sites) than for nitrate (*r* = 0.657, *r* = 0.505, and *r* = 0.236 at NF, SHA, and TTP, respectively). Increasing the depth of the mobile sensor at TTP reduced the amplitude of the diurnal nitrate pattern, while it did not seem to affect the measurements at SHA. However, the effect at SHA might be masked by the influence of rainfall events. At NF, the depth of the mobile sensor was reduced and the orientation was changed in a way that the measurement window was horizontal instead of at a 45° angle. This led to a reduction in the amplitude of the diurnal signal in both DOC and nitrate, as well as a shift in the phase of DOC. Changing the sensor orientation at TTP did not seem to affect the measurements of nitrate, although a slight phase shift was observed on the last day of measurements. Patterns for DOC measured by the mobile and fixed sensor did not correspond well when the orientation was changed, although the correlation coefficient was still relatively high (*r* = 0.725).

## 4. Discussion

### 4.1. Variations in Diurnal Patterns

The amplitudes of diurnal patterns for nitrate observed in our data were similar to those observed in temperate and subtropical rivers in the US, Spain, and the UK (0.01 to 0.15 mg N L^−1^) [2,18,38,39], but were smaller than those observed in a Mediterranean headwater stream (1.5 mg N L^−1^) [40] and other rivers in the UK (0.4–0.6 mg N L^−1^) [30,31]. A review on the diurnal cycling of DOC reports that changes by more than 100% of the background concentration have often been observed [17], which is much higher than the amplitude of the patterns measured in our study (<0.2 mg C L^−1^, background concentrations of 1.3–4.6 mg C L^−1^). Similar to our study, other authors report that diurnal patterns mainly occurred during low flow conditions and on dry days [18,20,38]. Other seasonal variations in diurnal patterns have been observed as well. Aubert and Breuer [21], for example, found seasonally varying diurnal patterns in nitrate concentration in a German headwater stream and attributed this to seasonal changes in evapotranspiration through riparian plant production. Additionally, changes in water depth, temperature, and related biotic activity have been used to explain seasonal variation in the amplitude and occurrence of diurnal patterns [41].

The shape and timing of diurnal patterns in nitrate and DOC concentrations in stream water are not consistent across streams and rivers worldwide. Differences are caused by variations in river metabolism, the extent to which biological activity influences solute concentrations, river size, and hydrological processes [42]. A typical pattern in nitrate concentrations exhibits a pre-dawn peak and afternoon minimum, driven by changes in the autotrophic uptake of nitrate [17]. These patterns are most obvious in undisturbed, forested streams with low nitrate concentrations and corresponds to patterns observed at NF with sensor A and B, at SHA with sensor C, at TTP with sensor B, and at OUT with sensors B and G. However, shifts in the timing of peaks and opposite patterns (such as measured with sensor F) have been observed elsewhere as well and were attributed to increased nighttime denitrification [41], hydrodynamic dispersion, and transient storage [38].

The typical diurnal pattern for DOC tends to be the opposite of that for nitrate, with a daytime peak due to autotrophic production and a nighttime minimum caused by heterotrophic consumption [17]. These patterns were only measured by sensor B at TTP and OUT and by sensor G at OUT (Figure 2). However, because DOC constitutes a large pool of different compounds, diurnal patterns caused by metabolic processes might not always be reflected in DOC concentrations [17,43]. A low amplitude of diurnal variations, the absence of clear patterns [20,44], shifts in the timing of minima and maxima [38], or multiple maxima and/or minima [18] could also be a consequence of the aggregation of multiple diurnal changes occurring upstream [42]. The resulting pattern, as a sum of these various signals, could obscure diurnal variation caused by biological processing at the measurement site. In our dataset, this could particularly apply to OUT, whereas clearer diurnal patterns are expected in the headwater streams with a smaller catchment area (NF, TTP, and SHA).

### 4.2. Explanatory Variables

The results from the studies discussed above show that spatial and temporal variations in biotic activity and hydrological conditions could, in theory, be responsible for the observed differences in diurnal patterns between sites and flow classes. However, the fact that different sensors recorded different patterns at the same site under the same flow conditions (Figure 1 and Figure 2), as well as the abrupt changes in diurnal patterns following a change in sensor position (Figure 3) strongly suggest that these patterns are not (solely) caused by natural processes. The lack of consistent changes in the observed patterns during the different treatments in the sensor comparison experiment makes it difficult to identify potentially interfering factors.

Because a change in sensor position or shading led to a change in diurnal pattern in most cases, the results imply that the amount of incoming solar radiation influences the measurements. Few studies using UV-Vis spectrophotometers report the orientation of the sensor, and little guidance is provided by the manufacturers. The choice for a certain sensor orientation is often based on reducing biofouling and the settlement of sediment on the sensor window [45,46,47], while shielding from incoming UV radiation is rarely mentioned by researchers, e.g., [19]. There are no studies on the potential influence of exposure to sunlight on in situ UV-Vis measurements. An effect of incident solar radiation could particularly explain patterns whereby deviations from the background concentration are mainly observed during daylight hours (e.g., sensor F, Figure 1). Even sensors of the same model could exhibit differences in the spectral responsivity of the detector, resulting in different responses to incoming background light. However, Figure 3 shows that the measurements by two parallel sensors do not only deviate from each other during daytime, but also when it is dark, indicating the potential relevance of other factors.

Optical components of the UV-Vis sensors can be affected by moisture, drift, and the aging of the components. However, the current set-up of the sensor comparison experiment did not allow us to test whether such sensor characteristics could have affected the measurements. Additionally, diurnal changes in stream water temperature, which are likely to be more pronounced during low and medium flow, could affect the electronic circuit inside the sensors and thus the measurements. Differences in the responses resulting from sensor characteristics could explain divergence in the patterns at any time of the day, as this is irrespective of incoming solar radiation.

Sensor accuracy can play a role as well, especially when the amplitude of the diurnal patterns is small [48]. Our data show that patterns are more reproducible when the background concentration is higher (e.g., nitrate at TTP, Figure 3a,b), as long as two sensors are installed in parallel at the same site. This is irrespective of whether the observed patterns are artefacts or not. The accuracy of UV-Vis sensors—as with any analytical instrument—is generally reduced when measuring close to the detection limit [49], which could result in higher variation in the measurements and therefore inconsistent patterns in the deviation from the background concentration. The sensor comparison experiment also showed that all sensors generally recorded similar larger sub-daily variations, caused by, for example, rainfall events. Very small variations in solute concentrations are hard to reproduce with grab samples due to the accuracy of both the sensor and the laboratory equipment. Furthermore, in case grab samples cannot be analyzed directly, transport and storage time can influence the solute concentrations [20,50]. Although two studies managed to use grab samples to verify diurnal patterns measured with in situ sensors [29,51], this verification method remains challenging.

### 4.3. Implications for the Use of In Situ UV-Vis Sensors

Based on the results of this study, we suggest that further effort is required to verify small-scale variations in solute concentrations measured by in situ UV-Vis sensors. Few studies report on field tests of UV-Vis sensors, e.g., [30,52], while field conditions can deviate significantly from the controlled laboratory conditions under which many sensors are tested during development. In the field, sensors are subjected to seasonal and diurnal temperature variations. In addition, the fouling of the sensor measurement windows can occur through the oxidation of iron or manganese by UV light [23] or biofouling [21,25,47,53]. Additionally, other substances in the water can interfere with the measurements, such as nitrite or bicarbonate [45]. Several studies mention that high turbidity values (>80 FTU [45], >300–450 FTU [54], >1000 NTU [52]) reduce the ability of the UV-Vis sensors to provide valid measurements for nitrate. This can be especially problematic in tropical regions with high soil erosion rates, where rainfall events result in short but sharp increases in turbidity. Due to differences in the water matrix, diurnal temperature fluctuations and turbidity, it could be possible that UV-Vis sensors behave differently in temperate versus tropical streams. Further testing of the validity of small, short-term changes in solute concentrations measured with in situ UV-Vis sensors should include a longer deployment of multiple sensors at one site. These could be either sensors of the same model or models produced by different manufacturers. Because rainfall seems to influence diurnal patterns, such testing should ideally occur under stable flow conditions. Furthermore, parameters that could potentially explain any artefacts, such as stream temperature, turbidity, discharge and incoming solar radiation, should be measured at the same time. In addition, controlled laboratory experiments should be performed to assess, for example, the influence of temperature and sensor-specific properties on measurements.

In case diurnal patterns measured with in situ UV-Vis are indeed artefacts, this has several implications for the use and interpretation of the high-resolution data. Lupon et al. [39] mention, for example, that diurnal cycling reduces the annual nitrogen load. This means that the use of datasets with artificial diurnal patterns could lead to the underestimation of annual loads. Furthermore, when grab samples are used to assess the overall validity of high-frequency in situ measurements or for calibration, the measured value at the time of sample collection might not represent the actual value. Nevertheless, numerous studies have successfully verified and calibrated in situ UV-Vis measurements using grab samples, e.g., [23,28,45,55].

Our data show that larger variations in solute concentrations, such as responses to rainfall events, can be reproduced by multiple sensors. Therefore, data collected by in situ UV-Vis sensors can be used to investigate longer term trends and seasonal variations, where minor sub-daily fluctuations are not as important. Additionally, event-based analyses, such as hysteresis analysis, e.g., [9,56], should not be affected by the findings of this study provided that the concentration change is larger than the measurement uncertainty and sensor accuracy. However, it remains good practice to verify long- and short-term changes in solute concentrations with independent measurements, such as grab samples. When UV-Vis sensors are used without further manual sampling, a second sensor run in parallel (at least for part of the monitoring period) would be good practice. This is particularly the case if the information contained in the high-resolution measurements is being studied in more detail, such as for the identification of sub-daily variation or the detection of potentially new, previously unforeseen patterns and processes.

## 5. Conclusions

Our effort to verify diurnal patterns by installing a second, mobile sensor at various sites showed that the small diurnal variations in nitrate and DOC concentrations measured at our study sites could only be partly reproduced. This implies that one should be cautious when inferring on the occurrence of small-scale biogeochemical processes and river metabolism. Although the causes for the differences in measured diurnal patterns could not be identified, these field observations are useful evidence to inform current and future users of in situ UV-Vis spectrometers on potential limitations of data use. We recommend that a thorough investigation is carried out to identify field conditions or sensor-specific properties that cause the patterns measured by different sensors to deviate. We therefore call for the scientific community to perform a further field comparison of multiple UV-Vis sensors and controlled laboratory experiments to address this issue, ideally in collaboration with sensor manufacturers. Until more is known, we recommend interpreting data recorded by a single UV-Vis sensor with caution for the analysis of diurnal patterns in solute concentrations, especially when the diurnal variations are of the same order of magnitude as sensor accuracy or resolution. Nevertheless, long-term high-resolution datasets are still valuable to advance our understanding of hydrological and biogeochemical processes in different climatic regions and ecosystems across the world.

## Figures and Tables

**Table 1:** Characteristics of the study catchments in the South West Mau region, Kenya. Mean annual precipitation is for 2015 to 2018 and the median, minimum, and maximum daily discharge are presented for November 2014 to October 2019.

Site	Land Use	Coordinates ^1^	Area [km^2^]	Elevation [m a.s.l.]	Mean Annual Precipitation [mm y^−1^]	Median (min., max.) Discharge [m^3^ s^−1^]
NF	Natural forest	0°27′47.591″ S, 35°18′32.046″ E	35.9	1954–2385	1894	0.52 (0.082, 5.79)
SHA	Smallholder agriculture	0°24′4.024″ S, 35°28′31.733″ E	27.2	2380–2691	1568	0.22 (0.014, 3.55)
TTP	Tea and tree plantations	0°28′34.917″ S, 35°13′17.220″ E	33.3	1786–2141	1810	0.37 (0.056, 3.82)
OUT	Mixed	0°28′59.548″ S, 35°10′54.557″ E	1021.3	1715–2932	1769	12.1 (1.36, 66.6)

^1^ Datum/projection: WGS1984 UTM Zone 36S.

**Table 2:** Deployment period of the mobile sensor for the sensor comparison experiment.

Site	Start Time	End Time	Treatment
NF	05-09-2017 11:20	28-09-2017 10:00	Parallel
	28-09-2017 11:10	02-10-2017 08:40	Shading
	02-10-2017 09:20	05-10-2017 11:00	Change depth + orientation
TTP	05-10-2017 13:30	23-10-2017 09:30	Parallel
	23-10-2017 09:40	26-10-2017 10:00	Change depth
	26-10-2017 10:20	30-10-2017 08:50	Shading
	30-10-2017 09:10	02-11-2017 11:20	Change orientation
SHA	10-11-2017 10:10	24-11-2017 09:40	Parallel
	24-11-2017 10:00	27-11-2017 11:00	Change depth
	27-11-2017 11:10	01-12-2017 10:00	Shading

**Table 3:** Breakpoints (*Q*_70_ and *Q*_30_) for the discharge classes, and the mean and standard deviation for the background concentration of nitrate and dissolved organic carbon for the four study sites (NF, SHA, TTP, and OUT) in the South West Mau region, Kenya.

Site	*Q*_70_ [m^3^ s^−1^]	*Q*_30_ [m^3^ s^−1^]	Nitrate [mg N L^−1^]			Dissolved Organic Carbon [mg C L^−1^]		
			Low Flow	Medium Flow	High Flow	Low Flow	Medium Flow	High Flow
NF	0.32	0.84	0.36 ± 0.13	0.42 ± 0.08	0.42 ± 0.09	2.58 ± 1.19	2.86 ± 1.45	2.93 ± 1.26
SHA	0.08	0.46	0.52 ± 0.12	0.89 ± 0.16	1.30 ± 0.26	4.56 ± 1.70	2.18 ± 0.92	1.39 ± 0.36
TTP	0.24	0.64	1.17 ± 0.28	1.74 ± 0.21	2.21 ± 0.29	3.16 ± 1.60	1.80 ± 0.96	1.29 ± 0.75
OUT	6.41	19.7	0.65 ± 0.19	0.84 ± 0.19	0.99 ± 0.19	3.51 ± 1.31	2.32 ± 1.10	2.02 ± 0.65

**Figure A2:**
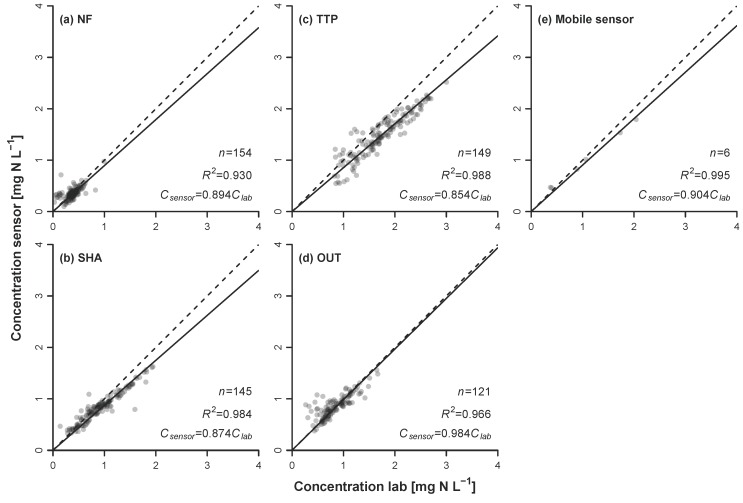
Calibration models for nitrate for the fixed sensors installed at (**a**) NF, (**b**) SHA, (**c**) TTP, and (**d**) OUT and for (**e**) the mobile sensor.

**Figure A3:**
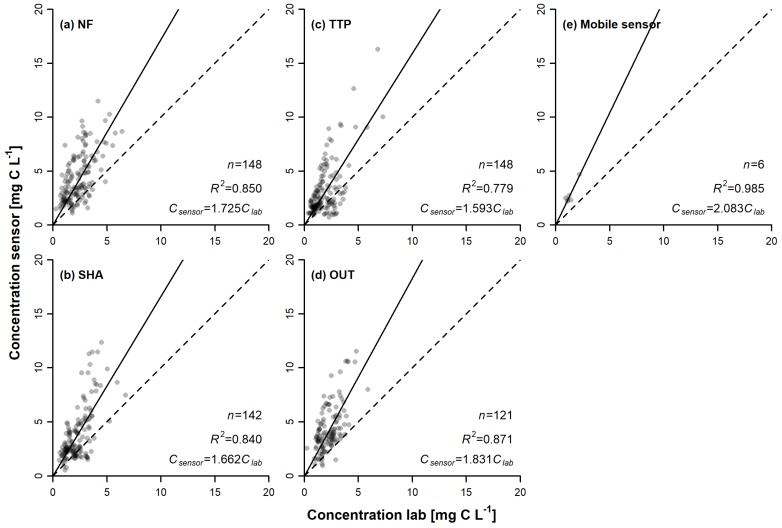
Calibration models for dissolved organic carbon (DOC) for the fixed sensors installed at (**a**) NF, (**b**) SHA, (**c**) TTP, and (**d**) OUT and for (**e**) the mobile sensor.

## Appendix A

Due to sensor failure, spectro::lyser sensors were replaced and repaired several times during the study period. In additions, sensors were rotated in May 2017 to test whether this would lead to changes in the observed diurnal variations in solute concentrations. Figure A1 gives an overview of the deployment period of each sensor.

## Appendix B

All sensors were calibrated using grab samples analyzed for dissolved organic carbon and nitrate. A linear regression model was developed for each site between laboratory and sensor values:

(A1) $$C_{sensor}=aC_{lab}$$

where *C_sensor_* is the solute concentration measured by the sensor in mg L^−1^ and *C_lab_* is the solute concentration measured in the grab sample analyzed in the laboratory in mg L^−1^. Coefficient *a* is the slope of the linear regression model. The reverse of the regression equation was applied to the full dataset:

(A2) $$C_{cal}=\frac{C_{sensor}}{a}$$

where *C_cal_* is the calibrated solute concentration. Figure A2 shows the regression models for nitrate and Figure A3 for dissolved organic carbon (DOC).
